# PlantLayout pipeline to model tissue patterning

**DOI:** 10.18699/VJ20.590

**Published:** 2020-02

**Authors:** M.S. Savina, V.V. Mironova

**Affiliations:** Institute of Cytology and Genetics of Siberian Branch of the Russian Academy of Sciences, Novosibirsk, Russia; Institute of Cytology and Genetics of Siberian Branch of the Russian Academy of Sciences, Novosibirsk, Russia Novosibirsk State University, Novosibirsk, Russia

**Keywords:** mathematical modeling, image analysis, polarity, morphogen, pattern, математическое моделирование, обработка изображений, полярность, морфоген, паттерн

## Abstract

To study the mechanisms underlying developmental pattern formation in a tissue, one needs to analyze the dynamics of the regulators in time and space across the tissue of a specific architecture. This problem is essential for the developmental regulators (morphogens) that distribute over the tissues anisotropically, forming there maxima and gradients and guiding cellular processes in a dose-dependent manner. Here we present the PlantLayout pipeline for MATLAB software, which facilitates the computational studies of tissue patterning. With its help, one can build a structural model of a two-dimensional tissue, embed it into a mathematical model in ODEs, perform numerical simulations, and visualize the obtained results – everything on the same platform. As a result, one can study the concentration dynamics of developmental regulators over the cell layout reconstructed from the real tissue. PlantLayout allows studying the dynamics and the output of gene networks guided by the developmental regulator in specific cells. The gene networks could be different for different cell types. One of the obstacles that PlantLayout removes semi-automatically is the determination of the cell wall orientation which is relevant when cells in the tissue have a polarity. Additionally, PlantLayout allows automatically extracting other quantitative and qualitative features of the cells and the cell walls, which might help in the modeling of a developmental pattern, such as the length and the width of the cell walls, the set of the neighboring cells, cell volume and cell perimeter. We demonstrate PlantLayout performance on the model of phytohormone auxin distribution over the plant root tip.

## Abstract (ru)

Для изучения механизмов, лежащих в основе формирования паттернов развития в тканях, необходим анализ динамики распределения регуляторов во времени и пространстве в тканях сложной структуры. Наибольшее значение это имеет в случае регуляторов развития (морфогенов), которые распределены в ткани неравномерно, образуя максимумы и градиенты, и регулируют клеточные процессы по-разному в зависимости от дозы. Настоящая работа посвящена описанию программного средства PlantLayout на языке MATLAB, которое облегчает исследование формирования распределений регуляторов в тканях различного строения. С его помощью можно построить двумерную структурную модель ткани, внедрить ее в математическую модель, описанную в терминах обычных дифференциальных уравнений, выполнить численные расчеты модели и визуализировать полученные результаты – всё на одной платформе. В результате можно изучать динамику работы генных сетей и изменения концентрации регуляторов в каждой клетке клеточного ансамбля, воспроизводящего строение реальной ткани. Исследуемые генные сети могут различаться для разных типов клеток. Одной из задач, которую решает PlantLayout в полуавтоматическом режиме, является определение ориентации клеточной стенки, что имеет значение в случае присутствия в ткани поляризованных клеток. Кроме того, PlantLayout позволяет автоматически определять другие качественные и количественные характеристики клеток и клеточных стенок, такие как длина и ширина клеточных стенок, площадь сечения и периметр клеток, которые могут помочь в моделировании паттернов распределения регуляторов развития, а также список соседних клеток для каждой клетки. В данной работе продемонстрирована эффективность разработанного программного средства PlantLayout применительно к моделированию распределения фитогормона ауксина в кончике корня растения.

## Introduction

Pattern formation requires the activity of developmental regulators to be controlled in time and space. This demands the application of computer modeling tools to predict the output from complex regulatory circuits and to understand if different developmental scenarios exist under certain conditions (Tomlin, Axelrod, 2007; Bellomo, Carbonaro, 2011). Developmental biology attracted much attention from specialists in mathematical modeling and computer sciences who built mathematical models to address fundamental problems in this field. One of the first mathematical models for tissue patterning formation was Turing’s reaction-diffusion model describing the interaction of two regulators: an activator and an inhibitor (Turing, 1952). This model helped understand the mechanisms of the pattern formation of the gap genes’ distribution during Drosophila melanogaster embryogenesis (Lacalli, 1990) and of the patterning of palatal ridges (Economou et al., 2012) or digits (Raspopovic et al., 2014) during mouse embryogenesis. Mathematical modeling of tissue patterning was also successfully used to study such processes as somitogenesis and neurogenesis for animals (Lewis, 2003; Shimojo, Kageyama, 2016) and phyllotaxis for plants (Jönsson et al., 2006; Smith et al., 2006). Nowadays, a number of professional tools and software have been developed to help the researchers in building more powerful computer models that take into account more experimental data. If the first mathematical models did not consider the cellular structure of the tissue at all, e. g. in Turing model (Turing, 1952), nowadays scientists are reducing the use of models with oversimplified rectangular tissues and start developing models on the realistic cell layouts.

According to the plant-image-analysis.org database, there are a large number of tools to do cell segmentation in twodimensional (2D) and three-dimensional (3D) analyses of tissue architecture (Lobet et al., 2013). Some of them are tissue-specific, for example, the plugin for ImageJ named Cefiler is intended for identification of cell files during wood anatomy analysis in 2D. Cefiler allows extracting data on the cell shape and the perimeter, the neighbors for each cell, the width of the cell walls (Brunel et al., 2012). ImageJ-plugin LSM-W2 is intended for wheat leaf structure analysis (Zubairova et al., 2019). The iRoCS Toolbox is a tool for 3D analysis of the root apical meristem of Arabidopsis thaliana (Schmidt et al., 2014). It allows identifying attachment of the cell to the definite tissue and measuring some quantitative characteristics like the volume of cell, radius, and position of the nuclei. Other tools can be used for different plant tissues and organs. DRACO-STEM is a pipeline for 3D segmentation (Cerutti et al., 2017). It allows getting only the topological structure of the tissue represented as a structural graph with vertices, edges, faces, and polyhedra as a node. MorphoGraphX is a free Linux application for the visualization and cell geometry analysis of organ surface in 3D (de Reuille et al., 2015). It allows performing the cell segmentation and time-lapse analysis of growth tissues. Balloon Plugin is a plugin for Fiji that allows extracting the lists of individual cell shapes and determining the cells being in contact with each other (Federici et al., 2012). CellSeT is a powerful software that can be used for the analysis of confocal images to provide quantitative data on the cell structure and expression intensity (Pound et al., 2012).

There are also several tools for mathematical modeling of plant tissues, for example VirtualLeaf (Merks et al., 2011), Cellzilla (Shapiro et al., 2013) and SimuPlant (Eckardt, 2014). On the one hand, these tools require the user to set the cell layout in a specific manner, they are not adapted to receive data from image processing programs. On the other hand, the image processing programs/tools have different data formats and their results require additional processing before being implemented into a mathematical modeling tool. For example, Band and the coauthors (2014) used three individual programs and an additionally developed script to build a mathematical model of phytohormone auxin distribution over the root apex. Specifically, they used CellSeT for image analysis of optical sections of the root apex from confocal microscopy, then, via an additional Python script, they imported CellSeT output into the OpenAlea simulation environment (Pradal et al., 2008) for creating the computational structural model representing a multicellular root geometry. Finally, OpenAlea output data was ready to start SimuPlant simulation (Eckardt, 2014).

The recent trend is that more biologists are eager to study the developmental processes of their interest using computational modeling methods. That demands the development of user-friendly software that helps biologists build the structural model on the basis of the experimental image, embed it then into a mathematical model and then perform numerical simulation on the same platform. Here we present the PlantLayout pipeline for MATLAB software for such purposes.

## Materials and methods

**Fig. 1.**
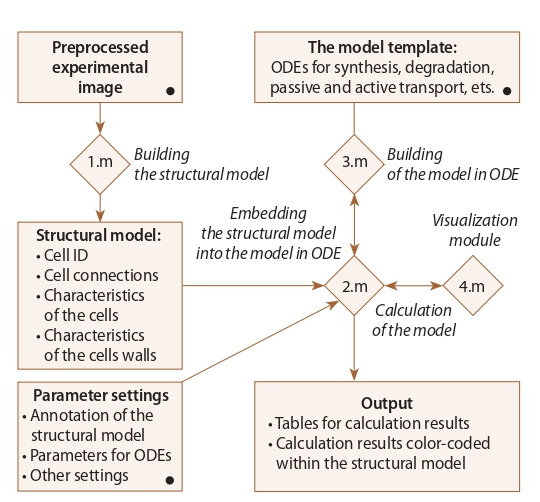
PlantLayout pipeline. Interactions between different parts of PlantLayout. Connected m-files are depicted by rhombuses. The procedures performed inside PlantLayout are written in italics. The points to be uploaded or edited by the user are marked with the black circles.

**PlantLayout pipeline.** PlantLayout is a pipeline built as four connected files (1.m–4.m) consisting of executable blocks in the MATLAB m-code programming language (Fig. 1). It uses MATLAB Image Processing Toolbox for creating twodimensional (2D) computational structural models of plant tissues and organs, embed them into a mathematical model in ordinary differential equations (ODE) and facilitate their numerical analysis. Most of the executable blocks inside PlantLayout files contain detailed descriptions and examples.

**The input data.** PlantLayout uses a preprocessed microscopic image as input data. One can either use third-party tools for image segmentation (e. g. CellSeT (Pound et al., 2012) or MorphoGraphX (de Reuille et al., 2015)) or draw the tissue scheme using the experimental image as a readout. In the first case, a manual correction might be in need. The second way might be also an option when one wants to study the influence of tiny anatomical changes.

**Building a 2D structural model.** PlantLayout applies bwconncomp function from MATLAB Image Processing Toolbox to identify all cells and cell walls as connected components within the preprocessed image (1.m in Fig. 1). The cells from the layout receive unique index numbers from 3 to N + 2 (where N is the number of cells). Herewith index 1 identifies the external environment, and index 2 identifies the part of the organ or tissue that is not included in the simulated cell layout. Every cell wall receives a couple of index numbers (i, j ), where i is the number of the mother cell and j is the number of the neighboring cell. PlantLayout automatically determines quantitative and qualitative characteristics for all cells and cell walls (see output data).

**An algorithm to define the cell wall orientation**

**Fig. 2.**
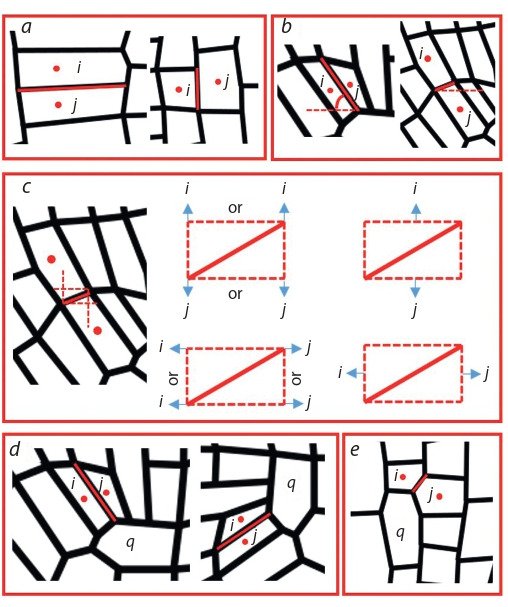
The rules for determination of the cell wall orientation in Plant- Layout. Representative examples of the cell wall orientation between the cells with the numbers i and j. a, determination using the maximum and the minimum coordinates for the cell wall; b, determination using the angle between the major axis of the characteristic ellipse and x-axis; c, determination using the smallest rectangle containing the cell wall; d, determination using the common neighbor cell with the number q when the cell walls with numbers (i, q) and ( j, q) have the same directions; e, determination using the common neighbor cell with number q when the cell walls with numbers (i, q) and ( j, q) have different directions.

We developed a special algorithm to determine the cell walls’ orientation. For this, several characteristics were calculated for the cells and cell walls considered as connected components. The first characteristic is the centroid of the region (cell or cell wall). The second is the smallest rectangle containing the region. The third is the characteristic ellipse that has the same normalized second central moments as the region. For the ellipse, the algorithm calculates the length of its major and minor axes and the angle between the x-axis and the major axis of the ellipse. Determination of the cell walls orientation (“top”, “bottom”, “right” and “left”) occurs according to the following algorithm:

- If the maximum and minimum coordinates of the cell wall (i, j ) lie between the centroids of the cells i and j vertically/ horizontally, then the orientation of the cell wall (i, j ) is “bottom”/“right” (Fig. 2, a).
- If the cell wall (i, j ) is vertical (the angle between the major axis of the characteristic ellipse and x-axis is more than 45 degrees) and the centroid of the cell j is to the right of the centroid of the cell i, then the orientation of the cell wall (i, j ) is “right”. If the cell wall (i, j ) is horizontal (the angle between the major axis of the characteristic ellipse and x- axis less than 45 degrees) and the centroid of the cell j is to the top of the centroid of the cell i, then the orientation of the cell wall (i, j ) is “top” (see Fig. 2, b).
- An alternative definition of orientation using the smallest rectangle containing the cell wall (i, j ). If the cell i is above the rectangle, and the cell j is below the rectangle, the orientation of the cell wall (i, j ) is “bottom”. The “top”, “right” and “left” orientations are defined in a similar way (see Fig. 2, c).
- If the orientation of the cell wall (i, j ) at points 1–3 is defined equally and unambiguously, then the orientation of the cell wall (i, j ) is determined.
- If there is an ambiguity in determining the orientation of the cell wall (i, j ), common neighbors of the cells i and j should be taken into account as described further.
- If the cell q is the common neighbor of the cells i and j and the cell walls (i, q) and ( j, q) are both vertical (“right” or “left”), then the cell wall (i, j ) is considered as perpendicular to them (“top” or “bottom”). If the cell walls (i, q) and ( j, q) are both horizontal (“top” or “bottom”), then the cell wall (i, j ) is considered as perpendicular to them (“right” or “left”). “Right” or “left” (“top” or “bottom”) orientation is determined by the relative position of the centroids of the cells i and j (see Fig. 2, d ).
- If the cell q is the common neighbor of the cells i and j and the cell wall (i, q) is vertical (“right” or “left”) and the cell wall (q, j ) is horizontal (“top” or “bottom”), it needs to compare the centroids positions of the cells i and j. If the vertical coordinates of the cell i centroid are more/less than the vertical coordinates of the cell j centroid and the orientation of the cell walls (q, j ) is “bottom”/“top”, then the orientation of the cell wall (i, j ) is “bottom”/“top”. If the horizontal coordinates of the cell i centroid are more/ less than the horizontal coordinates of the cell j centroid and the orientation of the cell walls (i, q) is “left”/“right”, then the orientation of the cell wall (i, j ) is “left”/“right” (see Fig. 2, e).
- If there is an ambiguity in determining the orientation of the cell wall (i, j ) using common neighbors, then manual correction should be used.

**Output 2D structural model**

PlantLayout provides the following quantitative and qualitative characteristics of the 2D cell layout taken from the preprocessed microscopic image: (1) the size of each cell (cross-sectional area and perimeter of the cell); (2) the size of each cell wall between any two adjacent cells (length and width); (3) the orientation of the cell walls (1, “top”; 2, “bottom”; 3, “right”; 4, “left”) for each cell. The quantitative characteristics of the cells are represented as column vectors, where the dimension of the vector corresponds to N + 2, and the indexes of the vector correspond to unique index numbers. The characteristics of the cell walls are represented by square matrices of dimension (N + 2), where the index (i, j ) corresponds to the cell wall with number (i, j ). If there is no cell wall between cells with indexes i and j, the value for the indexes (i, j ) and ( j, i) is set to zero. In the case of quantitative characteristics (the length and width of the cell wall) the values for the indexes (i, j ) and ( j, i) are equal. In the case of a matrix describing the orientation of the cell wall the values for the indexes (i, j ) and ( j, i) take the opposite values (“top”- “bottom” or “left”-“right”).

**The framework for embedding the structural model into the mathematical model**

At the next step, PlantLayout transfers the 2D structural model into the framework for mathematical modeling in MATLAB. The framework consists of two connected .m files for: (1) description of ODE system (3.m in Fig. 1), (2) the interrelations between the 2D structural model and the mathematical model in ODEs (2.m in Fig. 1). The 3.m file contains the frame for creating ODEs that describe the dynamics in the concentration levels for the substances of interest (variables). PlantLayout provides the examples for ODEs in general forms (the forms are different for the substances that spread through the cell layout and that do not move through the cell membrane). Only the functions that describe the appearance/disappearance of the modeling substance (e. g. synthesis, degradation, conjugation, oxidation, etc.) should be described for the substances that do not move. PlantLayout provides examples of functions in a common view to describe these processes according to Michaelis–Menten kinetics and Generalized Hill function method (Likhoshvai, Ratushny, 2007). In addition, the functions describing passive or active transportation from cell to cell should be described for the substances that are able to move through the plasma membrane. Based on Fick’s law of diffusion, the passive transport is proportional to the difference in substance concentrations between the cells. The active transport is described according to the mass action law, depending on the concentration of the carriers and the concentration of the moving substance. It is possible for the user to build the functions that differ from the default ones.

The 2.m file allows embedding the quantitative and qualitative data on the tissue structure received from the microscopic image into the mathematical model and performing its numerical analysis. This file consists of several executable blocks for different purposes (here the user can not only define a parameter set but also make the model adjustment). E. g. the user can specify the cells of different types (that possess different variables), transfer the length units to μm and mm. Some additional parameters required for the mathematical model can be calculated here. For example, in the case of diffusible substances, the flow through the cell wall of certain orientation might be specified. PlantLayout provides recommendations to describe such additional parameters and settings.

Finally, this 2.m file contains the frame for numerical calculation of the resulting mathematical model in ODEs. By default, PlantLayout uses MATLAB function ode15s that is a variable order solver based on the numerical differentiation formulas (NDFs) and the backward differentiation formulas (BDFs, also known as Gear’s method) (Shampine, Reichelt, 1997; Shampine et al., 1999). But the user may also apply other ODE solvers.

**Visualization of the model simulation results**

PlantLayout provides an option for visualization of the numerical simulation results on the base of the 2D structural model (4.m in Fig. 1). The concentration values calculated for every cell of the tissue are encoded using a color scale and visualized within the cell layout. The user can use any suitable colormap for visualization of the calculation results.

## Results and discussion

**PlantLayout pipeline to model tissue patterning**

We developed the PlantLayout pipeline in MATLAB software to facilitate the studies on the mechanisms of tissue patterning when the user needs to consider both the tissue architecture and the dynamics of the developmental regulators. The pipeline application is especially effective when the developmental regulator is able to move from cell to cell, generating gra-dients, maxima, and minima that are instructive for tissue patterning.

**Fig. 3.**
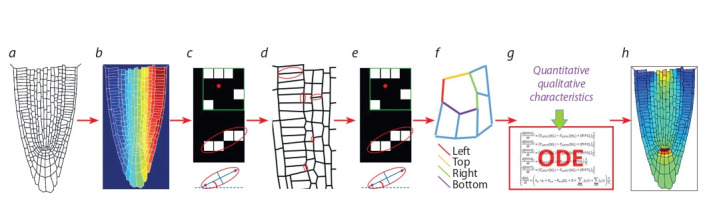
Representation of the PlantLayout pipeline on the example of building the model for the study of phytohormone auxin distribution within the plant root tip. a, pre-processed confocal image of the root tip; b, identifying the cells as connected components and getting unique indexes for each cell; c, quantification of cells features; d, identification of the cell walls between each two cells as connected components; e, estimation of the quantitative and qualitative characteristics for the cell walls; f, definition of the cell wall orientations; g, embedding the quantitative and qualitative characteristics of the 2D structural model into the mathematical model; h, steady state solution of the mathematical model, auxin distribution in the plant root tip.

PlantLayout consists of three steps: (1) creation of the 2D structural model of a tissue/organ, (2) embedding the mathematical model in ODE into the structural model, (3) numerical calculation and visualization of the results. As input data, the pipeline uses the preprocessed microscopic image of a 2D section of a tissue or organ (see Methods; Fig. 3). First, the pipeline determines the cells and the cell walls as connected components and gives them unique IDs. Then it automatically extracts for each cell and each cell walls their quantitative and qualitative features, such as the IDs of the neighboring cells, the cell size, the length and orientation of the cell wall (see Methods).

At the second step, the data on the anatomical structure of the tissue in the form of vectors and matrices is conveyed to the framework for the mathematical model construction. Here the user should (1) describe the ODEs for the processes he/ she wants to study; (2) annotate the cells in case 2D tissues consists of different cell types; (3) define which ODEs are embedded into the cells of a specific type; (4) define the initial data for the variables; (5) define parameters set (see Methods; Fig. 1). Note that some of the model parameters might depend on the structural characteristics of the 2D cell layout, e. g. the cell area and perimeter, the length, and width of the cell wall between each two cells.

Once the computational model has been ready, one can start its numerical analysis. Importantly, the results of numerical simulations can be visualized using the 2D structural model (see Fig. 3, h).

**PlantLayout deals with anisotropy in tissue patterning**

PlantLayout is especially relevant to the study of the developmental regulators that are able to move from cell to cell, e. g. morphogens. Some developmental regulators have an anisotropic distribution that cannot be described by simple diffusion. To deal with it, we built-in PlantLayout with an algorithm for automatic definition of the cell walls’ orientation.

Unlike other software products for creation of the structural models (e. g. CellSeT (Pound et al., 2012), Balloon Plugin (Federici et al., 2012)), PlantLayout defines the orientation of the cell walls in the 2D image (“top”, “bottom”, “left”, “right”). This feature is also relevant to the tissues with polar cells, in which cell walls have unequal functions. For example, when the cell transports a regulator inside at the “top” side, outside at the “bottom” side and does not transport them at the lateral sides. This is a characteristic behaviour for e. g. vascular cells in plants and intestinal epithelial cells in animals.

**PlantLayout application example**

To demonstrate the PlantLayout performance, we build with its help the 2D structural model that represents the longitudinal section of the root tip of Arabidopsis thaliana (see Fig. 3, a–f ). We embedded the structural model into an existing mathematical model of the phytohormone auxin in the root tip (Mironova et al., 2012; Hong et al., 2017). Auxin is a major regulator of plant root morphogenesis that distributes polarly via active transport and regulates cell dynamics depending on concentration. The initial model describes how auxin redistributes over the rectangular cell layout imitating the root tip tissue by passive and active auxin-regulated transport. Description of the mathematical model is beyond this study. In Fig. 3, h, you can see the steady state solution for auxin distribution that was generated with PlantLayout from uniform initial data and with the parameters set inherited from the initial model (Hong et al., 2017). The steady state solution has the specific auxin maximum inside the root tip that was also self-generated in the rectangular models (Mironova et al., 2012; Hong et al., 2017), but it gives more details about auxin concentrations in the cells of different types that did not exist in the rectangular model.

## Conclusion

Here we present the PlantLayout pipeline for MATLAB software which helps studying the molecular-genetic mechanisms of tissue patterning in the 2D tissue context. PlantLayout allows easily going from the preprocessed microscopic image to the mathematical modeling of tissue patterning through all stages at the same platform (see Figs. 1, 3), and this procedure can be repeated many times. Thus, a typical scenario of PlantLayout use is an analysis of a regulatory core output on the tissues of different architectures, e. g. to study the differences between wild type and mutant. Although PlantLayout was developed to study plant tissue patterning, it can be successfully applied for other organisms, if the cells in the tissue do not migrate, have tight connections and their shape can be approximated by a convex polygon.

## Conflict of interest

The authors declare no conflict of interest.
